# Genome wide gene expression regulation by HIP1 Protein Interactor, HIPPI: Prediction and validation

**DOI:** 10.1186/1471-2164-12-463

**Published:** 2011-09-26

**Authors:** Moumita Datta, Ananyo Choudhury, Ansuman Lahiri, Nitai P Bhattacharyya

**Affiliations:** 1 Crystallography and Molecular Biology Division, Saha Institute of Nuclear Physics, 1/AF Bidhan Nagar, Kolkata 700 064, India; 2 Department of Biophysics, Molecular Biology and Bioinformatics, University of Calcutta, 92 Acharya Prafulla Chandra Road, Kolkata 700009, India; 3 Wits Bioinformatics, University of the Witwatersrand, Johannesburg, Private Bag 3, 2050 Wits, South Africa

## Abstract

**Background:**

HIP1 Protein Interactor (HIPPI) is a pro-apoptotic protein that induces Caspase8 mediated apoptosis in cell. We have shown earlier that HIPPI could interact with a specific 9 bp sequence motif, defined as the HIPPI binding site (HBS), present in the upstream promoter of *Caspase1 *gene and regulate its expression. We also have shown that HIPPI, without any known nuclear localization signal, could be transported to the nucleus by HIP1, a NLS containing nucleo-cytoplasmic shuttling protein. Thus our present work aims at the investigation of the role of HIPPI as a global transcription regulator.

**Results:**

We carried out genome wide search for the presence of HBS in the upstream sequences of genes. Our result suggests that HBS was predominantly located within 2 Kb upstream from transcription start site. Transcription factors like CREBP1, TBP, OCT1, EVI1 and P53 half site were significantly enriched in the 100 bp vicinity of HBS indicating that they might co-operate with HIPPI for transcription regulation. To illustrate the role of HIPPI on transcriptome, we performed gene expression profiling by microarray. Exogenous expression of HIPPI in HeLa cells resulted in up-regulation of 580 genes (p < 0.05) while 457 genes were down-regulated. Several transcription factors including CBP, REST, C/EBP beta were altered by HIPPI in this study. HIPPI also interacted with P53 in the protein level. This interaction occurred exclusively in the nuclear compartment and was absent in cells where *HIP1 *was knocked down. HIPPI-P53 interaction was necessary for HIPPI mediated up-regulation of *Caspase1 *gene. Finally, we analyzed published microarray data obtained with post mortem brains of Huntington's disease (HD) patients to investigate the possible involvement of HIPPI in HD pathogenesis. We observed that along with the transcription factors like CREB, P300, SREBP1, Sp1 etc. which are already known to be involved in HD, HIPPI binding site was also significantly over-represented in the upstream sequences of genes altered in HD.

**Conclusions:**

Taken together, the results suggest that HIPPI could act as an important transcription regulator in cell regulating a vast array of genes, particularly transcription factors and at least, in part, play a role in transcription deregulation observed in HD.

## Background

HIPPI (HIP1 Protein Interactor) was identified by Gervais and co-workers as an interacting partner of Huntingtin Interacting Protein 1 (HIP1). HIPPI interacts with HIP1 through its pseudo death effector domain (pDED) and the resulting heterodimer recruits Procaspase8 and activates it thereby inducing Caspase8-mediated apoptosis [1]. Subsequently we have shown in detail the downstream pathways of Caspase8 activation leading to cell death in neuronal and non-neuronal cells [2]. Such non-receptor mediated induction of apoptosis may play a role in Huntington's disease (HD) pathogenesis. HD is an autosomal dominant neurodegenerative disease caused by the expansion of poly glutamine (Q) stretch at the N-terminus of the protein Huntingtin (HTT) [3]. HIP1 interacts strongly with wild type HTT but the interaction is feeble with mutant HTT [4]. Thus, in the diseased condition where one of the HTT allele is mutated, the freely available HIP1 in the cytoplasm may undergo heterodimerization with HIPPI, which in turn activate Caspase8 [1]. In addition, exogenous expression of HIPPI also increases the expression of Caspase1, Caspase3, Caspase7 and Caspase8 in cells as well as it induces truncation of Bid and release of AIF from mitochondria [2]. Although the protein lacks any conventional DNA binding domain, it is shown to interact *in vitro *and *in vivo *with a specific 9 bp DNA sequence 5'-AAAGACATG-3' present at the putative promoter of *Caspase1 *gene and positively regulate its transcription. Using various variants of the motif, we observed that HIPPI binds with the motif (AAAGA[G/C]A[A/C/T][T/G]) [5-7].

Structural analysis of HIPPI failed to detect any known protein domain except for a pseudo death effector domain (pDED) and a myosin like domain (MLD). The protein does not contain nuclear localization signal (NLS) and therefore is expected to translocate to nucleus via some carrier protein. Earlier study from our lab demonstrates that HIP1 acts as the carrier for HIPPI. The HIPPI-HIP1 heterodimeric protein complex formed in cytoplasm enters the nucleus through the NLS present at the C terminus of HIP1 [8] and assembles on the putative promoter of *Caspase1 *gene to regulate its transcription [9].

The role of HIPPI as a transcription regulator of *Caspase1 *thus imparts a new function to the protein. It is, therefore, important to identify other genes that could be regulated by HIPPI and the downstream effect of such regulation in cells. Gene expression regulation is a complex phenomenon in which several transcription factors work in concert to bring about the alteration. Thus, it is also important to look for the involvement of other cellular transcription factors in HIPPI mediated transcription regulation. In an attempt to decipher HIPPI's role as a general transcription regulator, in the present communication, we carried out genome wide search for the presence of HIPPI binding sites in the upstream sequences of genes coded by the human genome. Using our in-house search tool, we also predicted other transcription factors that might co-operate with HIPPI. Finally, to study the global changes in gene expression by HIPPI in cell, microarray experiment was carried out. One of our aims was to investigate the role of HIPPI in Huntington's disease pathogenesis. For this, we analyzed the gene expression data obtained by Hodges et al., [10]. Subsequent analysis of all the data could establish HIPPI's role as a global transcription regulator as well as its involvement in the deregulation of genes in HD.

## Methods

### Development of search tools

In our study, the computational analysis of transcription factor binding site (TFBS) enrichment was performed using an in house custom made tool. A set of 245 position weight matrices (PWMs), corresponding to known vertebrate TFBSs were obtained from the publicly available TRANSFAC database [11]. Binding sites of two other TFs, HIPPI [6] and NRSF/REST [12,13] were also included in this list. 10 Kb upstream sequences (5' of TSS) of all Human (version GrCh37) and Mouse (version NCBIM37) protein-coding genes were retrieved using the Biomart utility provided by Ensembl web server (http://www.ensembl.org/biomart/martview). The Human-mouse homolog genes were also determined using the same Biomart utility of the Ensembl web server.

PWM search (including both strands) with various similarity cutoff levels were performed to identify the location of each putative TFBS in all human and mouse 10 Kb upstream sequences. Enrichment of TFBS was analyzed among a target and a background set of upstream sequences using two different statistical tests. Depending on the source organism of the target genes (human or mouse) the entire collection of non-redundant human/mouse upstream sequences was used as the background set. The binding sites for which the cumulative hypergeometric P-value or the Chi square test P-values was less than 0.05 were considered to be enriched in the target upstream sequence set.

### Transcription factor binding site analysis

To identify the genes that harbor the 9 bp HIPPI binding motif (AAAGA[G/C]A[A/C/T][T/G]) in their upstream promoter region, we searched the 10 Kb upstream sequences of all the genes in the human genome using the in-house matrix search tool (MST). The Matrix combination search tool (MCST) was used to identify co-occurrence of HIPPI binding sites and other transcription factor binding sites in the gene promoters within a defined distance (100 bp). Functional classification of selected genes was performed by data retrieval tool. Functional classes having hypergeometric p value (corrected using Benjamini and Hochberg method) less than 0.05 were selected.

Additionally, TF binding site analysis and GO analysis on subset of genes were carried out using online tool Genecodis v2.0 [14,15].

### Dataset preparation for Huntington's disease microarray analysis

Microarray data obtained from HD patients' brain sample [10] were analyzed to study the involvement of HIPPI in HD pathogenesis. In the original study, Hodges et al., analyzed the mRNA expression level in 44 HD patients (Vonsattel Grades 0-4) and 36 age and sex matched controls using Affimetrix HG-U133A and HG-U133B arrays. Expression profiling was done for caudate, cerebellum and two cortical areas, BA4 (motor cortex) and BA9 (prefrontal association cortex). A statistical criterion p < 0.001 was used to obtain the differentially expressed genes. For our analysis, we downloaded the gene expression data for caudate, the brain region mostly affected in HD and sorted them based on the statistical significance (p < 0.05). We used false discovery rate (FDR, Benjamini and Hochberg method) for multiple testing of the array data and genes having corrected p value less than 0.05 were selected for analysis. The gene ids of the differentially regulated genes were converted to their corresponding Ensembl ids using Ensembl Biomart and analyzed using the in house search tools as described above.

### Antibodies and other reagents

Geniticin, Hygromycin, and anti Beta-actin (A2228, clone AC-74, Lot number: 107K4791) antibody were obtained from Sigma Chemicals (MO, USA). The anti-mouse and anti-rabbit secondary antibodies conjugated with horseradish peroxidase and protein A agarose beads were purchased from Bangalore Genei, India. Anti HIPPI antibody (ab5205-100, Lot number: 63362) was purchased from Abcam, USA. Anti P53 (IMG 80061) and anti Caspase1 antibodies (IMG-804-4, Lot number: AB093004A) were purchased from Imgenex, USA. Anti HIP1 antibody (NB300-204, 1B11, Lot number: A) was purchased from Novus Biologicals. Immobilon-P Transfer membrane was from Millipore, USA, *Taq polymerase *from Bioline, USA, and restriction enzymes (BamHI, SalI, and HindIII) were from Promega, USA. Protease inhibitor cocktail was purchased from Roche, USA. TRIZOL reagent was obtained from Invitrogen, USA. Microarray labeling kit was from GE Healthcare. Other molecular biology grade fine chemicals were procured locally.

### Construction of clone

Construction of GFP-Hippi has been described earlier [2]. In brief, full length human *HIPPI *was cloned in pEGFPC1 vector between SalI and BamHI sites. Full length *p53 *cloned in pCMV neo bam vector was kindly gifted by Dr. Susanta Roychoudhury, Indian Institute of Chemical Biology, Kolkata.

### Cell culture and transfection

HeLa and Neuro2A cells were routinely grown in MEM (HIMEDIA, India) supplemented with 10% fetal bovine serum (Biowest, USA.) at 37°C in 5% CO_2 _atmosphere under humidified condition. Transfection of cells was performed using Lipofectamine 2000 (Invitrogen, USA). Unless otherwise mentioned, for single transfection experiment 2.5 μg (60 mm plate) or 5 μg (100 mm plate) of DNA constructs as well as 5 μl or 10 μl of Lipofectamine 2000 respectively were used. After 24 h, transiently transfected cells were checked for transfection efficiency by monitoring GFP expression under fluorescence microscope and were used for experiments. Transfection efficiency varied from 70-90%.

### Knockdown of HIP1 and p53 in HeLa cells

Knock down of *HIP1 *in HeLa cells by sequence specific siRNA has been described earlier [9]. Briefly, DNA sequences 779-ACCGCTTCATGGAGCAGTTTA-799 of human *HIP1 *(gi|38045918|ref|NM_005338.4|) were used for designing the siRNA using the online software from GenScript (https://www.genscript.com/ssl-bin/app/rnai). The complete sequence inserted into the expression vector pRNATin-H1.2/Hygro was 5'-TAAACTGCTCCATGAAGCGGTTTGATATCCGACCGCTTCATGGAGCAGTTTATTTTTTCCAA-3' (designated Hip1Si) with termination signal and appropriate restriction site linkers (*BamH1 *and *HindIII*, not shown) and an insert for loop formation (underlined). The clones were checked by restriction digestion. *HIP1 *siRNA clone was transfected in HeLa cells using Lipofectamine 2000 (Invitrogen, USA) following manufacturer's protocol. Stably transfected cells were selected by Hygromycin resistance. Knock down of *HIP1 *in these cells was confirmed by western blot analysis using anti HIP1 antibody.

For knock down of *HIP1 *in Neuro 2A cells, the same siRNA construct and protocol was used as described above.

For generating *p53 *knock down HeLa cell line, pSuppressorNeo p53 plasmid DNA containing *p53 *siRNA construct (Imgenex, USA, catalog no. IMG 803) was used.

### Microarray study

For microarray study, total RNA from cell was extracted using RNeasy Mini Kit (Qiagen, USA) following manufacturer's protocol. RNA samples were quantified by measuring the absorbance at 260 nm and purity was determined using the OD^260^/OD^280 ^ratio. For cDNA preparation and labeling of the cDNA with fluorescent dyes Cy3 and Cy5, 10 μg of total RNA was reverse transcribed and labeled using CyScribe Post-Labeling Kit (GE Healthcare). Equal concentration of differentially labeled control and test samples were mixed and hybridized to the whole genome human 40 K array (Ocimum Biosolutions, India). Hybridization was carried out over night at 42°C in Hybstation (Genomic Solution). Hybridized array was scanned using GenePix Pro 4200 A scanner. Each experiment was repeated four times, twice with swapping the dye.

### Analysis of Microarray data

Analysis of microarray data was carried out by GenePix Pro 6.0 soft ware. The GenePix Result file (GPR) generated for each array were then transferred to Acuity 4.0 soft ware for statistical analysis. Significantly altered probes were identified by student's t test (p < 0.05). Using the same software, the correction for multiple testing was carried out. The detail protocol and array data has been submitted to Gene Expression Omnibus database (http://www.ncbi.nlm.nih.gov/geo) GEO accession nos. GSE26115 and GSE26116).

### Semi quantitative RT-PCR

Total RNA was extracted from cells using TRIZOL reagent (Invitrogen, USA). Two μg RNA was reverse transcribed using random hexamer primer (Fermentas, USA) and MuLv-Reverse transcriptase (Fermentas, USA). Semi quantitative RT-PCR was carried out using Red *Taq *DNA polymerase (Bioline, USA). Expression of beta actin was taken as endogenous control. Densitometry of the bands was done using Image Master VDS software (Amarsham Biosciences, UK). The primer sequences used for the gene expression analysis are listed below.

CBP-F: 5' TTGCAGAGGTCTTTGAGCAGG 3'

CBP-R: 5' ATCGCGAGGAATGGTACACAG 3'

GNG10-F: 5' TGGTAGAGCAGCTCAAGTTGG 3'

GNG10-R: 5' TCAGAGTAAAGCACAGGATCTAGG 3'

CREB3L2-F: 5' CCCTTCACCCACATTACCAC 3'

CREB3L2-R: 5' TCATTTCCAGAGGAGGTTCC 3'

CACNG1-F: 5' TGTCCCTCGGGAAGAAGAG 3'

CACNG1-R: 5' CAGGCAAAGGACCAGGAGTA 3'

VTI1A-F: 5' GCAAATTGGTCAGGAGATGTT 3'

VTI1A-R: 5' GATGGTGATGACCACGATGA 3'

NKX2-5-F: 5' ACCCAGCCAAGGACCCTA 3'

NKX2-5-R: 5' GCGTGGACGTGAGTTTCAG 3'

C/EBPβ-F: 5' GAGCAAGGCCAAGAAGACC 3'

C/EBPβ-R: 5' AGCTGCTCCACCTTCTTCTG 3'

ID1-F: 5' GCTCTACGACATGAACGGCTGT 3'

ID1-R: 5' GTTCCAACTTCGGATTCCGAGT 3'

CCL5-F: 5' CTGCTGCTTTGCCTACATTGC 3'

CCL5-R: 5' CCGAACCCATTTCTTCTCTGG 3'

ITPR1-F: 5' ACCTGCTGGTGGCGTTTTT 3'

ITPR1-R: 5' TGAGAGGCAGGAAGAGCAGAGA 3'

### Sub-cellular fractionation, Immunoprecipitation and Western Blot analysis

Methods for sub-cellular fractionation, immunoprecipitation and Western blot analysis were essentially the same as described earlier [9]. Briefly, cells grown in 100 mm Petri dishes were washed with ice cold PBS and harvested at 300 g for 3 min at 4°C. Cytosol was extracted using cytosol extraction buffer (50 mM Tris-Cl pH 7.5, 10 mM NaCl, 2 mM EDTA, 1 mM PMSF and 1X protease inhibitor cocktail, 0.25% NP-40). The nuclear pellet was then suspended in nuclear IP buffer (50 mM Tris-Cl pH 7.5, 150 mM NaCl, 2 mM EDTA, 1 mM PMSF and 1X protease inhibitor cocktail) followed by repeated freezing and thawing and centrifugation at 13,000 g for 20 min at 4°C. The extracts were then incubated with anti P53 antibody (1:100 dilution) and BSA soaked protein-A agarose beads and kept overnight at 4°C under continuous rotating condition. Next day the immunoprecipitated complex was collected by centrifugation at 1000 g for 2 min at 4°C. Beads were washed and boiled with SDS gel loading buffer and were subjected to western blot using anti HIPPI and anti P53 antibodies.

For immunoprecipitation assay using whole cell extract, cell lysis was carried out using co-immunoprecipitation buffer (50 mM Tris-Cl pH 7.5, 15 mM EDTA, 100 mM NaCl, 0.1% Triton X-100 and PMSF with 100 μg/ml final concentrations). Beta actin was used as internal loading control. Integrated optical density (IOD) of each band was calculated using Image Master VDS software (Amarsham Biosciences, UK). Whenever necessary, IOD was normalized with that of the loading control.

### Statistical analysis

All the experiments (except microarray experiment) were done for three times. Statistical analysis, mainly unpaired t test was carried out using the on-line software GraphPad QuickCalc available at http://www.graphpad.com/quickcalcs/ttest1.cfm

## Result

### Genome wide search for the presence of HIPPI binding sites in the upstream of coding genes

**Figure 1:**
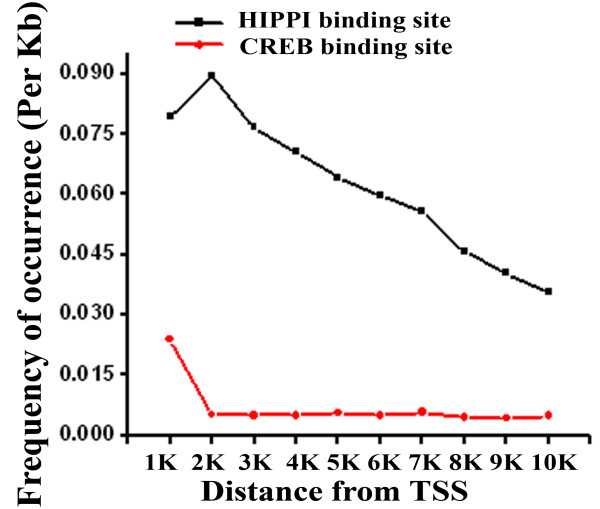
**Distribution of HIPPI binding site (HBS) along the 10 Kb upstream sequences of all the genes in the human genome**. Distance was calculated taking the transcription start site (TSS) as '0'. Frequency of occurrence of HBS per kb distance was plotted against the distance from TSS. CREB binding site distribution (Red line) along the same distance was shown to compare the pattern of distribution.

To identify genes that could be regulated by HIPPI, we carried out a genome wide search for the presence of 9 bp HIPPI binding motif (AAAGA[G/C]A[A/C/T][T/G]) as described earlier [6] in the upstream sequences of genes coded by the human genome. Initially, we started with 10 Kb upstream sequences and then narrowed it down to 1 Kb upstream. Using a score 1.0 (perfect match) MST (please see the materials and methods) predicted that 1872 (7.9%) out of total 23,607 genes contained HIPPI binding site (HBS) within 1 Kb upstream of their coding strands. This number increased to 14,586 (61.8%) when we searched 10 Kb upstream sequences. The distribution of HBS along the 10 Kb upstream of genes starting from the transcription start site (TSS, taken as position '0') in the window of 1 Kb is graphically represented in Figure 1. Result showed that majority of the genes contained the binding site within 2 Kb upstream and frequency decreased at a longer distance from TSS. As HIPPI is a non-conventional transcription regulator, we therefore compared this distribution pattern with another known transcription factor CREB (Figure 1). The binding sites for CREB were mainly concentrated within 1 Kb upstream of genes and showed similar pattern to that of HIPPI. As HBS was mainly enriched within 2 Kb upstream from TSS, we took those genes bearing HBS within their 2 Kb upstream promoters (3988, Additional file 1) and subsequently analyzed them by Gene Ontology (GO) for functional classification. The genes showed involvement in diverse cellular functions. Molecular functions like receptor activity (GO:0004872), sequence-specific DNA binding (GO:0043565), transcription factor activity (GO:0003700), transcription regulator activity (GO:0030528), chromatin binding (GO:0003682) and biological processes like signal transduction (GO:0007165), regulation of transcription, DNA-dependent (GO:0006355), translation (GO:0006412), synaptic transmission (GO: 0007268), positive regulation of transcription from RNA polymerase II promoter (GO:0045944), DNA damage response, signal transduction resulting in induction of apoptosis (GO:0008630), etc. were significantly enriched category (Table 1 and Additional file 2).

**Table 1:** GO Molecular Function and Biological Process analysis of the genes having HBS within 2 Kb upstream sequence

Molecular functions						
**GO ID**	**GO details**	**Data set frequency (%)**	**Background frequency (%)**	**p value (unadjusted)**	**p value (corrected)**	**Gene name**

GO:0004872	Receptor activity	9.84549	5.767347	5.71E-20	1.10E-17	Transforming growth factor, beta receptor 1, glutamate receptor, ionotropic, N-methyl D-aspartate 2D, gamma-aminobutyric acid (GABA) B receptor, 2

GO:0043565	Sequence-specific DNA binding	4.132231	2.602547	2.88E-07	9.29E-06	cAMP responsive element binding protein 1, Activating transcription factor 2, CCAAT/enhancer binding protein (C/EBP), gamma, POU class 4 homeobox 2

GO:0003700	Transcription factor activity	6.324111	4.527836	2.17E-06	6.00E-05	CREB/ATF bZIP transcription factor, cAMP responsive element binding protein 1, activating transcription factor 2, Signal transducer and activator of transcription 4, Transcription factor AP-2 beta

GO:0030528	Transcription regulator activity	3.162055	1.967883	4.25E-06	0.00010965	Inhibitor of DNA binding 1, dominant negative helix-loop-helix protein, POU class 4 homeobox 2, HMG-box transcription factor 1

GO:0003682	Chromatin binding	1.401365	0.758189	0.0001005	0.001620659	CREB binding protein, RE1-silencing transcription factor, polymerase (DNA directed), alpha 1, catalytic subunit, SWI/SNF related, matrix associated, actin dependent regulator of chromatin, subfamily e, member 1

**Biological process**						

GO:0007165	Signal transduction	14.94077	8.710653	6.47E-29	3.07E-26	Protein kinase, cAMP-dependent, regulatory, type II, beta, CREB binding protein, cAMP responsive element modulator, Transforming growth factor, beta receptor 1, cAMP responsive element binding protein 1, Caspase 1, apoptosis-related cysteine peptidase

GO:0006355	Regulation of transcription, DNA-dependent	9.70577	5.865315	1.04E-16	9.88E-15	CREB binding protein, Nuclear transcription factor Y, alpha, Nuclear factor of activated T-cells, cytoplasmic, calcineurin-dependent 4, cAMP responsive element binding protein 1, Cyclin-dependent kinase inhibitor 2A

GO:0006412	Translation	3.018724	1.682498	1.67E-07	7.21E-06	Eukaryotic translation initiation factor 6, WD repeat-containing protein 46

GO:0007268	Synaptic transmission	1.337409	0.694297	0.000101	0.001916701	Synapsin I, Neurexin 2, Amyloid beta (A4) precursor protein-binding, family A, member 1, Glutamate decarboxylase 1 (brain, 67 kDa), Gamma-aminobutyric acid (GABA) B receptor, 2

GO:0045944	Positive regulation of transcription from RNA polymerase II promoter	2.216278	1.478042	0.001084	0.01355529	Nuclear transcription factor Y, alpha, Transforming growth factor, beta 3, CREB binding protein, SMAD family member 4, Thyroid hormone receptor, beta,

GO:0008630	DNA damage response, signal transduction resulting in induction of apoptosis	0.22927	0.068152	0.005672	0.04954832	Methyl-CpG binding domain protein 4. Ataxia telangiectasia mutated. CHK2 checkpoint homolog (S. pombe)

### Enrichment of other transcription factor binding sites in the vicinity of HIPPI binding site

**Figure 2:**
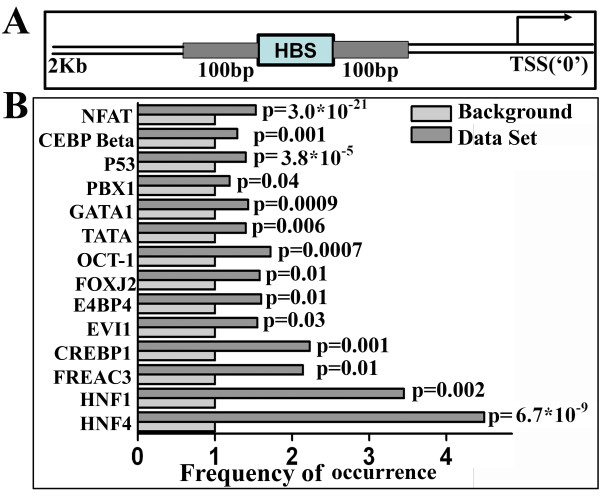
**Co-occurrence of HBS and other transcription factor binding sites in the genome**. A. Schematic representation of the region searched for the co-occurrence of HBS and other transcription factor binding sites. Hundred (100) base pair region on either side of HBS (grey shaded) were taken into consideration. B. Transcription factors whose binding sites were significantly enriched (p < 0.05) in the 100 bp region on either side of HBS in the 2 Kb upstream of genes. Data set represents the set of genes in the human genome having HBS within 2 Kb upstream regions. The occurrence of a particular motif within 100 bp distance of HBS in the data set was divided by the occurrence of that motif in the background and the ratio was plotted against the TF. The p value in the figure represents the significance level of enrichment for the transcription factor.

Generally transcription factors act in combinatorial manner to regulate target gene expression [16] and it is expected that binding sites for those transcription factors that work in concert should co-occur within a close distance. Thus, to identify the transcription factor partners in HIPPI mediated transcriptional regulation, we searched for the co-occurrences of other transcription factor binding sites within 100 bp distance on either side of HBS in the upstream sequence of the genes (Figure 2a). Since HBS was mainly centered within 2 Kb upstream regions, we confined our search with those genes containing HBS within 2 Kb upstream. Using a cut off score 0.9 for the second transcription factor, 14 TFs were found to be significantly overrepresented (p < 0.05) within 100 bp on either side of HBS (Figure 2b). These TFs include Nuclear factor of activated T cells (NFAT), CCAAT enhancer binding protein beta (C/EBP beta), Tumor protein 53 (P53), pre-B-cell leukemia homeobox 1 (PBX1), GATA binding protein 1 (GATA1), TATA box binding protein (TBP), POU class 2 homeobox 1 (OCT1), Forkhead box J2 (FOXJ2), nuclear factor, interleukin 3 regulated (E4BP4), runt-related transcription factor 1 (RUNX1/EVI1), cAMP response element binding protein 1 (CREBP1), Forkhead box C1 (FOXC1/FREAC3), HNF1 homeobox A (HNF1) and Hepatocyte nuclear factor 4, alpha (HNF4). These TFs, therefore, may have role in HIPPI mediated transcription regulation.

### Alteration of gene expression by HIPPI: microarray study

**Figure 3:**
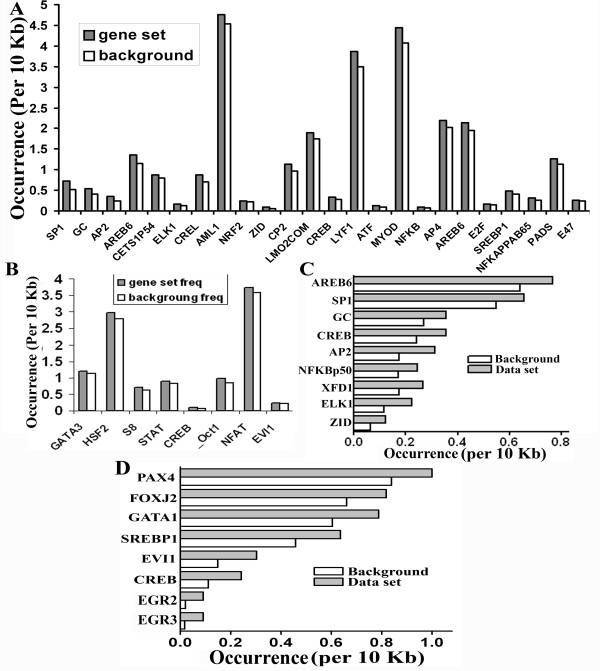
**Enrichment of transcription factor binding sites in the upstream of genes altered in microarray study**. A. Genes those were up-regulated by HIPPI in HeLa cells were analyzed for the presence of transcription factor binding sites in their upstream. The occurrence of a particular binding motif in the set of up-regulated genes was compared to the background occurrence of that motif and plotted against the TF. B. Same analysis as in A was done with the genes that were down-regulated by HIPPI in HeLa cells. C. Same analysis as in A was done with the up-regulated genes those were common in the two sets of microarray. D. Same analysis as in A was done with the down-regulated genes those were common in the two sets of microarray.

To explore the effect of HIPPI on cellular transcriptome, we carried out gene expression profiling by microarray. HeLa cells exogenously transfected with GFP-Hippi construct (designated as 'HeLa-Hippi') was taken as test sample while HeLa cells transfected with empty GFP vector (designated as 'HeLa-Gfp') served as control and relative level of transcripts were compared between these cells by microarray. Among 39,936 spots in the array we observed up-regulation of 580 genes (p < 0.05) while 457 genes were down regulated (p < 0.05, Table 2, Additional file 3). These set of genes were then analyzed for the presence of transcription factor binding sites in their upstream. In contrary to our expectation, HBS was not significantly enriched in the upstream of either up or down-regulated genes. But several other transcription factors including specificity protein 1 (Sp1), Activator protein 2 (AP2), C-Rel, NF-kappaB p65 (relA), E47 etc were significantly (p < 0.05) enriched within 10 Kb upstream of the up-regulated genes (Figure 3a) while the down-regulated genes harbored binding sites for OCT1, NFAT, EVI1, HSF2 etc (Figure 3b). Binding sites for CREB was enriched in both up and down-regulated genes. TF binding site analysis was also carried out using a second method. The online tool Genecodis v2.0 [14,15] was used for this purpose. Along with the TFs obtained from our tool, Genecodis predicted the enrichment of P53 in the upstream of up-regulated genes (Additional file 4). Functional classification of these genes by GO revealed their involvement in various molecular functions and biological processes (Table 3 and Additional file 5). Transcription factor activity (GO:0003700), chromatin binding (GO:0003682), transcription co-activator activity (GO:0003713), transcription repressor activity (GO:0016564), sequence-specific DNA binding (GO:0043565) were some of the significantly enriched molecular function and positive regulation of transcription, DNA-dependent (GO:0045893), negative regulation of transcription from RNA polymerase II promoter (GO:0000122), translation (GO:0006412) were some of the significantly enriched biological processes for the up-regulated genes while down-regulated genes were enriched in the molecular functions like transmembrane receptor activity (GO:0004888), transcription repressor activity (GO:0016564), transcription co-repressor activity (GO:0003714) and biological processes like synaptic transmission (GO:0007268), signal transduction (GO:0007165), negative regulation of transcription from RNA polymerase II promoter (GO:0000122) etc. It is worthy of mentioning that some of these molecular functions like transcription factor activity, chromatin binding, sequence-specific DNA binding and biological processes like signal transduction, regulation of transcription, DNA-dependent, synaptic transmission were already predicted when we performed genome wide analysis of HBS containing genes (Table 1 and Additional file 2). The genes possessing HIPPI binding sites among the deregulated genes were analyzed separately for their involvement in cellular functions. We found that among the up-regulated genes, 342 genes contained HBS within 10 Kb upstream sequence and they were enriched in molecular functions like RNA polymerase II transcription factor activity (GO:0003702), transcription factor activity, (GO:0003700) and biological processes like regulation of transcription (GO:0045449) (Additional file 6). In case of the down-regulated genes, HBS was present within 10 Kb upstream sequence for 259 genes and they were enriched in biological processes like signal transduction (GO:0007165), synaptic transmission, glutamatergic (GO:0035249) etc. (Additional file 6). Again, some of these GO terms and some related GO terms were already found to be enriched when we performed genome wide analysis of HBS containing genes in the first section. Our experimental data, therefore, confirmed our predictions.

**Table 2:** Altered genes in Microarray Study

Experiment	No. of probes altered	No. of probes up-regulated	No. of probes down-regulated
HeLa/Hippi	1037	580	457

Hip1Si/Hip1SiHi	1649	990	659

Common	123	90	33

**Table 3:** Functional classification of the altered genes in microarray study by Gene Ontology

Up-regulated						
**Molecular function**						

**GO ID**	**GO details**	**Data set frequency (%)**	**Background frequency (%)**	**p value (unadjusted)**	**p value (corrected)**	**Gene name**

GO:0003700	Transcription factor activity	9.881422925	4.527835754	2.14E-07	3.54E-05	Cbp/p300-interacting transactivator, with Glu/Asp-rich carboxy-terminal domain, 2, NK2 transcription factor related, locus 5 (Drosophila), CCAAT/enhancer binding protein (C/EBP), beta, CREB binding protein

GO:0003702	RNA polymerase II transcription factor activity	2.56916996	0.575030881	7.44E-06	0.000531586	Forkhead box E1 (thyroid transcription factor 2), Transcription elongation regulator 1

GO:0016564	Transcription repressor activity	2.766798419	0.72837245	8.03E-06	0.0011916	Peroxisome proliferator-activated receptor delta, RE1-silencing transcription factor, NADH dehydrogenase (ubiquinone) 1 alpha subcomplex, 9, 39 kDa

GO:0003682	Chromatin binding	2.56916996	0.758188866	2.16E-05	0.004496337	NK2 transcription factor related, locus 5 (Drosophila), CREB binding protein, RE1-silencing transcription factor

GO:0003713	Transcription co-activator activity	2.56916996	0.796524258	3.49E-05	0.006663331	Transcription elongation regulator 1, junction mediating and regulatory protein, p53 cofactor, CREB binding protein

GO:0043565	Sequence-specific DNA binding	5.335968379	2.602547174	3.57E-05	0.01057545	v-rel reticuloendotheliosis viral oncogene homolog A (avian), CCAAT/enhancer binding protein (C/EBP), beta, NK2 transcription factor related, locus 5 (Drosophila)

**Biological process**						

GO:0000122	Negative regulation of transcription from RNA polymerase II promoter	3.29218107	1.086169442	9.09E-05	0.01248966	SIN3 homolog B, transcription regulator (yeast), RE1-silencing transcription factor

GO:0006355	Regulation of transcription, DNA-dependent	10.08230453	5.865314989	0.000119	0.01575762	Y box binding protein 1, POU class 2 homeobox 2, NK2 transcription factor related, locus 5 (Drosophila), CCAAT/enhancer binding protein (C/EBP), beta

GO:0045893	Positive regulation of transcription, DNA-dependent	1.851851852	0.442986753	0.000161	0.02283294	Cbp/p300-interacting transactivator, with Glu/Asp-rich carboxy-terminal domain, 2, Dual-specificity tyrosine-(Y)-phosphorylation regulated kinase 1B

GO:0045944	Positive regulation of transcription from RNA polymerase II promoter	3.703703704	1.478042339	0.000366	0.02283294	Zinc finger and BTB domain containing 38, CCAAT/enhancer binding protein (C/EBP), beta, CREB binding protein, Tumor necrosis factor receptor superfamily, member 1A

GO:0006412	Translation	3.909465021	1.682497764	0.000502	0.03143108	Eukaryotic translation initiation factor 1B, Eukaryotic translation initiation factor 6

**Down-regulated**						

**Molecular functions**						

GO:0004888	Transmembrane receptor activity	3.140096618	0.694296546	6.83E-06	0.00066934	G protein-coupled receptor 123, Toll-like receptor 6

GO:0016564	Transcription repressor activity	2.415458937	0.72837245	4.91E-05	0.02326809	MYB binding protein (P160) 1a, Runt-related transcription factor 1; translocated to, 1 (cyclin D-related)

GO:0003714	Transcription co-repressor activity	1.93236715	0.621885249	0.000116133	0.0479129	Nuclear receptor interacting protein 1, Histone deacetylase 9

**Biological process**						

GO:0007165	signal transduction	18.29896907	8.71065298	1.50E-09	1.06E-06	Ataxia telangiectasia mutated, Somatostatin receptor 2, Protein kinase, cAMP-dependent, regulatory, type II, alpha, Nuclear factor of kappa light polypeptide gene enhancer in B-cells inhibitor, beta

GO:0007268	synaptic transmission	2.835051546	0.694296546	4.92E-07	0.005691273	Neurexin 2, Potassium channel, subfamily K, member 3, Somatostatin

GO:0000122	negative regulation of transcription from RNA polymerase II promoter	3.350515464	1.086169442	2.23E-06	0.01540901	Nuclear receptor interacting protein 1, SP100 nuclear antigen, POU class 3 homeobox 3

Since nuclear localization of HIPPI is HIP1 mediated [9], we next carried out microarray experiment with *HIP1 *knocked down HeLa cells (designated as Hip1Si). Empty GFP vector transfected Hip1Si cells (designated as Hip1Si-Gfp) were used as controls while Hip1Si cells transfected with GFP-Hippi construct (designated as Hip1SiHippi) were used as test. Here, we observed alteration of 1652 genes of which 990 were up regulated and 662 were down regulated (p < 0.05, Table 2). The level of alteration of the genes in the two sets of microarray (HeLa-Gfp/HeLa-Hippi and Hip1Si-Gfp/Hip1SiHippi) was compared. It was expected that the genes that were regulated by HIPPI would show a diminished level of alteration in the second case as *HIP1 *knock down results in cytoplasmic accumulation of HIPPI [9]. We arbitrarily chose 10% as the cut off values for the ratios of expression of genes altered by HIPPI in the presence or reduced expression of HIP1. Such comparisons revealed that 90 genes up-regulated by HIPPI in HeLa cells were decreased in cells where *HIP1 *was knocked down. Similarly 33 genes that were decreased by exogenous expression of HIPPI in HeLa cells with endogenous HIP1 were increased in *HIP1 *knocked down cells. We assigned these 123 genes as the potential targets of HIPPI-HIP1 mediated transcriptional regulation and termed them as 'common set'. The lists of these genes are shown in the Additional file 3.

The common set of genes was analyzed for the enrichment of transcription factor binding sites. Binding motifs of ZID (p = 0.02), ELK1 (p = 0.003), XFD1 (p = 0.02), NFKappaB p50 (p = 0.04), AP2 (p = 0.0009) CREB (p = 0.01), GC (p = 0.04), Sp1 (p = 0.02) and AREB6 (p = 0.007, Figure 3c) were enriched in the upstream of up-regulated genes while down-regulated genes contained binding sites for EGR3 (p = 0.02), EGR2 (p = 0.03), CREB (p = 0.03), EVI1/RunX1, (p = 0.02), SREBP1 (p = 0.03), GATA1 (p = 0.02), FOXJ2 (p = 0.04) and PAX4 (p = 0.003, Figure 3d). Here again HBS was not enriched. The reason for this could be the fact that HIPPI is a non conventional transcription factor and was also found to regulate several transcription factors like CREB binding protein (CBP), neuron restrictive silencing factor (NRSF) etc. Therefore it is expected that many genes altered in presence of HIPPI could be due to its secondary effect mediated through the regulation of some of the other crucial transcription factors.

### Validation of subset of genes altered in microarray study

**Figure 4:**
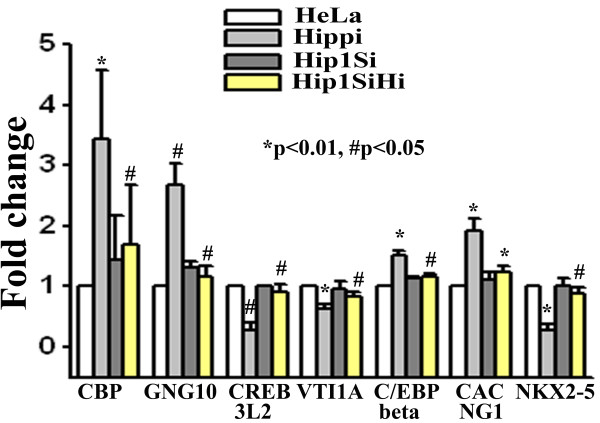
**Relative expression level of some of the altered genes in microarray study determined by RT-PCR**. Expression of beta actin was taken as control. Error bar represents standard deviation (n = 3). For each gene, expression level in 'Hippi' was compared with expression level in 'HeLa' and that in 'Hip1SiHi' with 'Hippi'. Significance levels of various pairs are indicated in the figure.

A set of genes altered in microarray experiments were validated using a second method like semi quantitative RT-PCR. From the list of altered genes in the two sets of microarray we randomly chose 10 genes and measured their level of expression in empty GFP vector transfected HeLa cells (designated as 'HeLa'), GFP-Hippi transfected HeLa cells (designated as 'Hippi'), empty GFP vector transfected *HIP1 *knocked down HeLa cells (designated as 'Hip1Si') and GFP-Hippi transfected *HIP1 *knocked down HeLa cells (designated as 'Hip1SiHi'). Among the 10 genes, expression pattern of 6 genes were consistent with that of microarray data (Figure 4). Exogenous expression of HIPPI in HeLa cells up-regulated the genes viz. Guanine nucleotide binding protein, gamma 10 (*GNG10*), CREB binding protein (*CBP*), CCAAT/enhancer binding protein, beta (*C/EBPβ*) and Calcium channel, voltage-dependent, gamma subunit 1 (*CACNG1*) while CREB3 like 2 (*CREB3L2*) and vesicle transport through interaction with t-SNAREs homolog 1A (yeast), (*VTI1A*) were down-regulated. However, exogenous expression of HIPPI in Hip1Si cells failed to alter the expression level of these genes indicating that both HIP1 and HIPPI were required for the transcriptional regulation of these genes. *NKX2-5 *was initially found to be up regulated in microarray study. But, semi quantitative RT-PCR showed that it was actually down-regulated by HIPPI (Figure 4). Three other genes chosen (viz. *TCERG1*, *RAX *and *RelA*) were unaltered in presence of HIPPI (data not shown).

These genes along with some others were also tested for their expression in presence of HIPPI in a second cell line like human neuroblastoma cells SHSY5Y. A comprehensive list summarizing all the single gene expression data is given in Additional file 7.

### Interaction of HIPPI with P53 in the nuclear compartment of cell and its effect on Caspase1 expression

**Figure 5:**
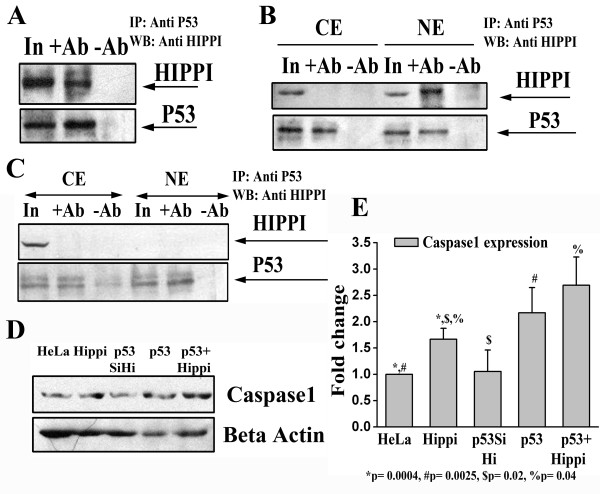
**HIPPI interacts with P53 in the nucleus and alters the expression of Procaspase1 in cell**. A. Co-immunoprecipitation of HIPPI with P53 from whole cell extract of Neuro2A cells. IP was carried out with anti P53 antibody and western blot was done with anti HIPPI antibody (*Upper panel)*. The same blot was stripped and reprobed with anti P53 antibody as IP control (*lower panel*). Lane 'In': input of immunoprecipitation, '+Ab': cell extract treated with anti P53 antibody, '-Ab': cell extract treated with IgG only. B. Co-immunoprecipitation of HIPPI with P53 from cytoplasm and nuclear compartment of Neuro2A cells. Cytoplasmic ('CE') and nuclear ('NE') fractions were separately immunoprecipitated with anti P53 antibody and detected with anti HIPPI antibody (*Upper panel*). The lane markings carry their usual meanings as described in A. C. Interaction of HIPPI with P53 in cytoplasm and nuclear compartment of N2AHip1Si cells. D. Western blot analysis for the detection of Procaspase1 expression in parental HeLa cells ('HeLa'), HeLa cells transfected with GFP-Hippi ('Hippi'), *p53 *knocked down HeLa cells exogenously transfected with GFP-Hippi ('P53SiHi'), HeLa cells transfected with full length *p53 *('P53') and HeLa cells co-transfected with full length *p53 *and GFP-Hippi ('P53+Hippi'). Beta actin was used as loading control. E. Bar diagram represents mean (n = 3) relative expression of Procaspase1 in different cells described in D above. IOD value of the Procaspase1 specific band was normalized with that of beta actin. Normalized IOD of 'HeLa' was considered to be 1.

In the earlier section, using MST we have predicted that binding site of P53 (half site) was enriched near the HBS for the genes containing HBS within their 2 Kb upstream. To check whether these two proteins could interact with each other we performed co-immunoprecipitation assay. Whole cell extract of Neuro2A cells (that express both P53 and HIPPI endogenously) showed positive co-immunoprecipitation of HIPPI with P53 (Figure 5a). To specifically locate the compartment where the complex was formed, we fractionated Neuro2A cells into cytoplasm and nucleus and observed that HIPPI co-immunoprecipitated with P53 only in the nuclear compartment (Figure 5b). To validate it further, we took *HIP1 *knocked down Neuro2A cells (N2AHip1Si) and studied the interaction of HIPPI with P53. Knock down of *HIP1 *has been confirmed by western blot analysis (Additional file 8). As HIP1 is the nuclear transporter of HIPPI, knock down of *HIP1 *restricts HIPPI in the cytoplasm and consequently no interaction between HIPPI and P53 was observed either in whole cell extract or in the nuclear compartment of N2AHip1Si cells (Figure 5c).

The interaction of HIPPI and P53 in the nuclear compartment of cell might have important role in HIPPI mediated transcription regulation. From our microarray data we have seen that P53 binding site was enriched in the upstream of up-regulated genes. To elucidate the functional relevance of this interaction we determined the level of Caspase1 expression in presence and absence of these two transcription factors. Human *Caspase1 *promoter contains a consensus P53 binding site at the position -117 to -98 from TSS [17]. It also contains a HIPPI binding site at the position -119 to -111 from TSS [6]. Thus to see the combinatorial effect of HIPPI and P53 on *Caspase1 *gene expression we transfected GFP-Hippi exogenously in *p53 *knocked down HeLa cells (Figure 5d and 5e designated as 'p53SiHi'). Knock down of *p53 *in HeLa cell was confirmed by western blot analysis [18]. These cells showed reduced expression of Caspase1 as compared to HeLa cells transfected with HIPPI (designated as 'Hippi', n = 3 p = 0.02) or P53 alone (designated as 'p53', p = 0.03). Co-transfection of HIPPI and P53 in HeLa cells (designated as 'p53+Hippi') resulted in even higher level of Caspase1 expression compared to the single transfected cells (p = 0.04). Thus in absence of P53, HIPPI alone could not increase *Caspase1 *gene expression; rather, the interaction with P53 appeared to be very important for HIPPI mediated up-regulation of the gene.

### HIPPI binding sites at the deregulated genes in the caudate of HD patients: possible role of HIPPI in HD

**Figure 6:**
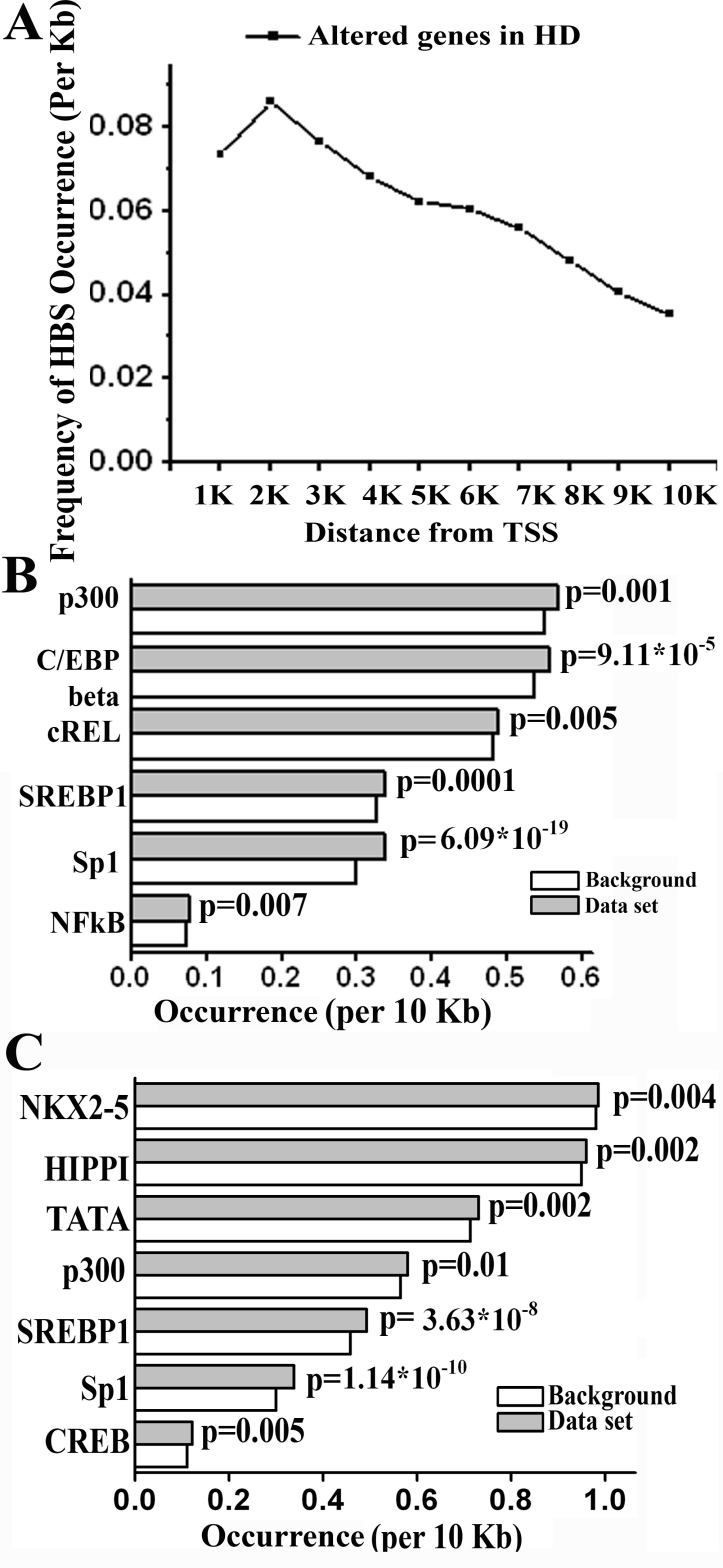
**Distribution of HBS and enrichment of transcription factor binding sites in the upstream of genes altered in HD**. A. HBS distribution along the 10 Kb upstream regions of the genes altered in microarray study carried out with HD patients. Distance was calculated taking the transcription start site (TSS) as '0'. Frequency of occurrence of HBS per kb distance was plotted against the distance from TSS as described in legend of Figure 1. B. Enrichment of transcription factor binding sites in the 10 Kb upstream of genes up-regulated in HD microarray. As described in Figure 3A, the occurrence of a particular binding motif in the set of up-regulated genes was compared to the background occurrence of that motif and plotted against the TF. The p value in the figure represents the significance level of enrichment for the transcription factor. C. Enrichment of transcription factor binding sites in the 10 Kb upstream of genes down-regulated in HD microarray as described above.

The involvement of HIPPI in HD pathogenesis is not well established but its ability to induce apoptosis through recruitment and activation of Caspase8 [1] and increased expression of Caspase1 [2,5-7,9] indicate that it may have some role in the disease. To elucidate it further, we first analyzed the distribution of HBS along the 10 Kb upstream promoters of the genes that were differentially expressed in a microarray experiment carried out with HD patients [10] (see materials and methods for details). The distribution was similar to that obtained with the whole genome (Figure 6a and Figure 1). Of the differentially regulated genes that do harbor HBS in their upstream, the majority of the sites in these genes were located within 2 Kb of TSS and the frequency of HBS occurrence reduced rapidly with increasing distance from TSS. Next we attempted to see whether HBS was significantly enriched in the upstream promoter of these genes. It was observed that HBS was significantly enriched only in the promoters (10 Kb) of down-regulated genes (p = 0.002, Additional file 9) but not in the up-regulated genes (p = 0.866). The altered genes were further analyzed to see whether any other transcription factor binding sites, particularly those TFs that are already known to be involved in HD, were enriched in their upstream promoters [19-24]. Transcription factors like P300, Sterol response element binding protein 1 (SREBP1), Sp1 were found to be significantly enriched in both up and down regulated genes but NFKappaB binding sites were enriched in up-regulated genes only while CREB and TATA box binding protein sites were enriched in down-regulated genes only (Figure 6b and 6c). This, in fact, correlated with the previous data that CRE (cAMP response element) mediated transcription is down-regulated in HD [19]. Next we functionally classified the altered genes using GO. Among the various molecular functions and biological processes enriched for the up-regulated genes in HD, we found that molecular function like transcription factor activity (GO:0003700), chromatin binding (GO:0003682), transcription co-activator activity (GO:0003713), sequence-specific DNA binding (GO:0043565), and biological processes like regulation of transcription (GO:0045449) were common to that obtained with genes up-regulated by HIPPI in the microarray study (Additional file 10). For genes down-regulated in HD, transcription co-repressor activity (GO:0003714) and synaptic transmission (GO:0007268) were within enriched category similar to that observed with genes down-regulated by HIPPI. Thus, the significant occurrence of HBS in the upstream of differentially expressed genes in HD and also the involvement of these genes in some of the functional categories that were perturbed by HIPPI in cell suggests that HIPPI may indeed play transcription regulatory role in the disease pathogenesis.

**Figure 7:**
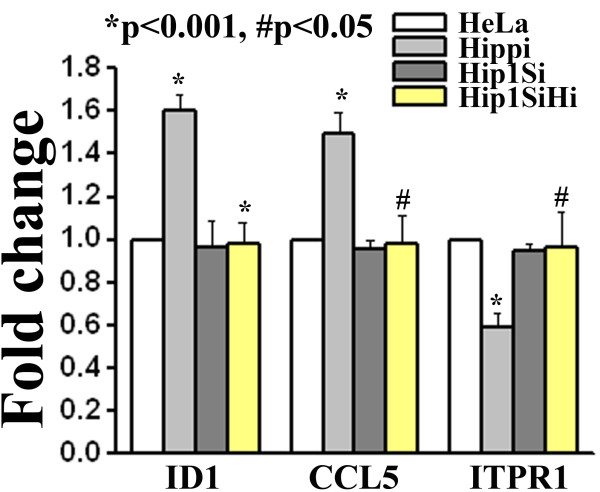
**Relative expression level of some of the genes altered in HD microarray study in presence of HIPPI**. Expression of beta actin was taken as control. Error bar represents standard deviation (n = 3). For each gene, expression level in 'Hippi' was compared with expression level in 'HeLa' and that in 'Hip1SiHi' with 'Hippi'. Significance levels of various pairs are indicated in the figure.

From the genes that were altered in HD microarray discussed above, we chose 4 genes viz. Chemokine (C-C motif) ligand 5 [*CCL5*, ENSG00000161570], Inhibitor of DNA binding 1 [*ID1*, ENSG00000125968], NADH dehydrogenase (ubiquinone) 1 alpha subcomplex, 10, 42 kDa [*NDUFA10*, ENSG00000130414] and Inositol 1,4,5-triphosphate receptor, type 1 [*ITPR1*, ENSG00000150995] which contained HBS in their upstream (Additional file 6). *CCL5 *and *ID1 *were up-regulated while *NDUFA10 *and *ITPR1 *were down-regulated in HD. We measured the expression level of these genes in presence of HIPPI. Both *CCL5 *and *ID1 *expressions were increased and *ITPR1 *expression was decreased in presence of HIPPI in HeLa cells (Figure 7) but remained unaltered in *HIP1 *knocked down HeLa cells. *NDUFA10 *expression was not detected in HeLa cells. CREB binding protein (*CBP*, ENSG00000005339) and RE1 Silencing transcription factor (*REST*, ENSG00000084093) were also found to be up-regulated in HD (Additional file 7). The alteration of these two genes in presence of HIPPI has already been demonstrated by microarray.

## Discussion

To investigate the role of HIPPI as a novel transcription regulator, we first took bioinformatic approaches to predict the targets of HIPPI and then tried to experimentally validate the predictions. Generally, transcription factor binding sites are enriched near the TSS and the frequency of occurrence of TFBS decreases as we move further upstream. The predominant occurrence of 9 bp consensus HIPPI binding site within 2 Kb upstream sequences of genes was similar to that of known transcription factor CREB (Figure 1). This indicates that HBS might be functionally active and regulate the expression of genes harboring HBS. Next, this set of predicted HIPPI targets were classified functionally using Gene Ontology to understand which biological processes or functions could be regulated by HIPPI in cells. The genes showed significant enrichment for functional categories related to transcriptional regulation, chromatin binding, signal transduction, receptor activity and induction of apoptosis (Table 1 and Additional file 2). It should be mentioned that HIPPI's role in apoptosis induction and transcription regulation has already been reported [1,2,5-7,9]. These functional categories were also found to be significantly over-represented when we analyzed our microarray data. Exogenous expression of HIPPI in HeLa cells resulted in alteration of ~ 1000 genes of which 580 genes were up-regulated and 457 genes were down-regulated (Table 2). Up-regulated genes were mainly enriched in the functional categories like transcription factor activity, transcription co-activator activity, chromatin binding, sequence specific DNA binding, positive regulation of transcription etc. (Table 3) while down-regulated genes were enriched in transcription repressor activity, transcription co-repressor activity, negative regulation of transcription, synaptic transmission etc. (Table 3). This suggests that HIPPI could participate in cellular transcription regulation machinery. To elucidate this property further, we carried out another set of microarray experiment where we blocked nuclear translocation of HIPPI by knocking down its transporter HIP1 [9]. We then compared the genes altered in the two sets of microarray. It was expected that genes that were transcriptional targets of HIPPI would show a reduced level of alteration in *HIP1 *knocked down cells. Results showed that 90 genes that were up-regulated by HIPPI in HeLa cells (having endogenous HIP1) were up-regulated to a lesser extent in *HIP1 *knocked down cells (Table 2 and Additional file 3). Similar was the case for 33 down-regulated genes. This subset of altered genes was thus under control of HIPPI-HIP1 mediated transcription regulation.

While analyzing our microarray data we found that multiple testing corrections yielded no statistically significant probes in the array. We, therefore, took the single gene validation approach. From our microarray data we chose 21 genes (unadjusted p value significant) and measured their expression in presence of HIPPI using a second method like semi quantitative RT-PCR or Real time PCR in HeLa (Figure 4) as well as in a second cell line (SHSY5Y, Additional file 7). Fifteen among the 21 genes (71%) showed similar trend as observed in microarray while one (NKX2-5) showed reverse trend. Five genes remained unaltered. Among these 16 validated genes, *REST *expression was found to be regulated by HIPPI in both neuronal and non-neuronal cells through interaction of HIPPI with *REST *promoter [25]. Further analysis of these 16 genes using GO revealed that they were enriched in functions like transcription factor activity, transcription co-activator activity, chromatin binding, sequence specific DNA binding and processes like regulation of transcription DNA dependent, positive and negative regulation of transcription from RNA pol II promoter etc. (Additional file 7) similar to those obtained with genome wide analysis (Table 1) and microarray analysis (Table 3). Thus, even though multiple testing of array data indicated lack of statistical significance for the altered probes, our single gene validation experiments suggest that HIPPI indeed interferes with these processes in cell.

Gene expression regulation is a complex phenomenon involving participation of several transcription factors in a co-operative manner [16]. To find out the transcription factor partners of HIPPI we carried out enrichment analysis for TF binding motifs within a fixed 100 bp distance from HBS (Figure 2a). Several transcription factor binding motifs, including NFAT, C/EBP beta, P53, TATA box binding protein, CREBP1 were over-represented in the 100 bp vicinity of HBS (Figure 2b). Presence of TBP, CREBP1 binding sites suggest that HIPPI may take part in basal transcription regulation machinery in cell. Among these TFs, C/EBP beta was found to be up-regulated by HIPPI (Figure 4 and Additional file 3). Binding motifs for GATA1, FOXJ2 and EVI1 have been enriched in the upstream of genes down-regulated by HIPPI (Figure 3c and 3d). Thus, interaction of HIPPI with these TFs may result in transcriptional repression. It was somewhat surprising that HBS was not enriched in the upstream of altered genes. One possible explanation for this could be the fact that HIPPI is not a conventional transcription factor (without DNA binding and transactivation domain). It is, therefore, possible that over-expression of HIPPI in cell increased its interaction with other cellular proteins rather than with DNA. Thus, the primary or direct targets of HIPPI constituted only a small fraction among the perturbed genes [26]. Rest of the altered genes constituted the secondary target set that were differentially expressed in response to the primary set or the changes in cellular physiology brought about by the primary set. For example, GATA3 and EVI1 were down-regulated by HIPPI in microarray study (Additional file 3). Binding sites for GATA3 and EVI1 were enriched in the upstream of down-regulated genes (Figure 3b). Thus a subset of genes down-regulated by HIPPI was effect of GATA3 or EVI1 down-regulation. This was also true for REST. Transcription repressor REST was up-regulated by HIPPI and consequently REST binding site was enriched in the upstream of down-regulated genes (Additional file 4). Similarly increased expression of CBP by HIPPI may result in increased histone acetylation leading to up-regulation of certain genes. It seems therefore important to analyze the primary set (those harboring HBS) separately from the bulk to understand direct transcription regulatory role of HIPPI. The similarities in GO classes between the primary set (Additional file 6) and those predicted from genome wide analysis (Additional file 2) indicates that these cellular processes were indeed perturbed by HIPPI.

HIPPI was found to interact with P53 in the protein level. This interaction took place in the nucleus (Figure 5b) and was dependent on the presence of HIP1, the nuclear transporter of HIPPI as HIPPI did not co-immunoprecipitate with P53 in the nuclear compartment of *HIP1 *knocked down cells (Figure 5c). This interaction was found to be necessary for HIPPI mediated up-regulation of *Caspase1 *gene expression. Human *Caspase1 *gene contains overlapping P53 binding site (position -117 to -98, [17]) and HIPPI binding site (position -119 to -111, [6]) in the promoter. It was observed that HIPPI could not induce Caspase1 expression in *p53 *knocked down HeLa cells (Figure 5d and 5e, comparing 'Hippi' with 'p53SiHi'). When both HIPPI and P53 were co-expressed, the expression of Caspase1 was strongly enhanced compared to the corresponding single transfected cells (Figure 5d and 5e, comparing 'Hippi' and 'p53' with 'p53+Hippi') indicating that both the transcription factors co-operated to regulate this gene expression. Such interaction may also play role in HD pathogenesis. It is observed that P53 protein level is increased in HD cell model [18]. Also HIPPI-HIP1 interaction is more in the diseased condition [1] which in turn may increase nuclear pool of HIPPI in cell. Therefore, the combinatorial effect of these two regulators will be more in the diseased condition.

Deregulation of transcription and induction of neuronal apoptosis are two of the major contributors to the pathogenesis of HD. Gervais and co-workers identified a novel non receptor mediated apoptotic cascade involving HIPPI and HIP1 that could operate in HD [1]. Work of Majumder et al., and Banerjee et al., imparted a transcription regulatory activity of HIPPI [2,5,6,9]. We therefore, tried to analyze the role of HIPPI in the disease pathogenesis. Microarray data obtained from HD patients [10] was analyzed for the enrichment of HBS in the upstream of altered genes and we found that HIPPI binding sites were enriched in the set of genes that were down-regulated in HD (Figure 6c). Additionally, the functional categories in which the altered genes in HD belong, bared similarity with the functions played by the HIPPI regulated genes. This includes transcription factor activity (GO:0003700), chromatin binding (GO:0003682), transcription co-activator activity (GO:0003713) for the up-regulated genes and transcription co-repressor activity (GO:0003714) and synaptic transmission (GO:0007268) for the down-regulated genes. We have shown that transcription co-activator CBP was up-regulated by HIPPI which can lead to transcriptional activation of a vast array of genes. Similarly, expression of ID1, an inhibitor that prevents DNA binding of basic helix-loop-helix transcription factors was up-regulated in presence of HIPPI which may cause repression of genes. Recently we have reported that HIPPI mediated transcriptional induction of *REST *plays an important role in repressing essential neuronal genes such as *BDNF *in HD cell model [25]. Thus HIPPI mediated transcriptional regulation may indeed contribute to the transcription deregulation observed in HD.

In summary, the manuscript addresses the potential transcription regulatory role played by HIPPI in cell. Using bioinformatic approach we identified a group of HIPPI regulated genes and their possible functions and subsequently validated them with microarray experiments. Although the lack of multiple testing corrections in array data is a limitation of this study, the similarities between functional classes of predicted and experimentally altered genes indicate perturbation of these functions by HIPPI in cells. We also predicted possible co-operativity of HIPPI with other transcription factors in the regulation of gene expression and have shown the synergistic role of HIPPI and P53 in regulation of Caspase1 expression. Finally, the involvement of HIPPI mediated transcription regulation is assessed in the context of Huntington's disease pathogenesis.

## Conclusion

In conclusion, the work presented here identifies new transcriptional targets of HIPPI. It also reveals new interaction between HIPPI and P53 and the effect of such interaction in cell. Finally the work predicts the involvement of HIPPI in HD pathogenesis. It would be of immense interest to investigate in detail, the transcription regulatory role played by HIPPI in Huntington's disease.

## Authors' contributions

MD and NPB conceptualized the entire work and wrote the paper. MD performed all the bioinformatic analysis and experimental works. AC and AL developed the search tools employed in the bioinformatic analysis and helped in data analysis and critical review of the paper. All authors read and approved the final manuscript.

## Supplementary Material

Additional file 1 **Genome wide occurrence of HBS within 2 Kb upstream sequences**. The file contains list of the genes that harbor HBS within their 2 Kb upstream promoter including the exact position and sequence of the motif matched. Click here for file

Additional file 2 **GO classification of genes having HBS within 2 Kb upstream**. The file contains significant molecular functions and biological processes in which the genes harboring HBS within 2 Kb upstream are involved. Click here for file

Additional file 3 **List of genes altered in microarray study**. The file contains details of the genes altered in HeLa-Gfp/HeLa-Hippi and Hip1Si-Gfp/Hip1SiHippi microarray study and also the list of genes in the common set including their fold change Click here for file

Additional file 4 **Transcription factor binding site analysis by Genecodis in the upstream of genes altered by HIPPI in microarray**. The file contains lists of transcription factors that are significantly enriched in the upstream of genes altered by HIPPI in microarray. Click here for file

Additional file 5 **GO classification of genes altered in HeLa-Gfp/HeLa Hippi microarray**. The file contains lists of molecular functions and biological processes significantly enriched for the genes altered by HIPPI in microarray study. Click here for file

Additional file 6 **GO classification of genes altered in HeLa-Gfp/HeLa Hippi microarray which contain HBS within 10 Kb upstream**. The file contains lists of molecular functions and biological processes significantly enriched for those genes that contain HBS within 10 Kb upstream and are altered by HIPPI in microarray study. Click here for file

Additional file 7 **Single gene expression analysis for genes altered in presence of HIPPI**. The file contains list of the genes whose expression was measured in presence of HIPPI in various cell lines using various methods. It also contains significantly enriched GO terms for these altered genes. Click here for file

Additional file 8 **Knock down of *HIP1 *in Neuro2A cells**. A. Western blot analysis for the detection of HIP1 (*upper panel*) in Neuro2A cells (designated as N2A) and N2A cells stably transfected with Hip1Si construct to knock down *HIP1 *(designated as N2AHip1Si). In the *lower panel*, the 42 KDa band represents beta actin as loading control. B: The bar diagram represents mean integrated optical densities of bands corresponding to HIP1 and normalized with that of the loading control beta actin. Level of significance (p values) is shown in the figure. Click here for file

Additional file 9 **List of genes harboring HBS within 10 Kb upstream among the genes altered in HD microarray**. The file contains list of the genes that are altered in HD microarray and also harbor HBS within their 10 Kb upstream promoter including the exact position and sequence of the motif matched. Click here for file

Additional file 10 **GO classification of genes altered in HD microarray study**. The file contains lists of molecular functions and biological Processes significantly enriched for the genes altered in HD microarray study. Click here for file
