# Supplementary figures and images for: Genome wide gene expression regulation by HIP1 Protein Interactor, HIPPI: Prediction and validation
Source: BMC Genomics. 2011 Sep 26;12:463. doi: 10.1186/1471-2164-12-463 (PMC3228557; doi:10.1186/1471-2164-12-463)

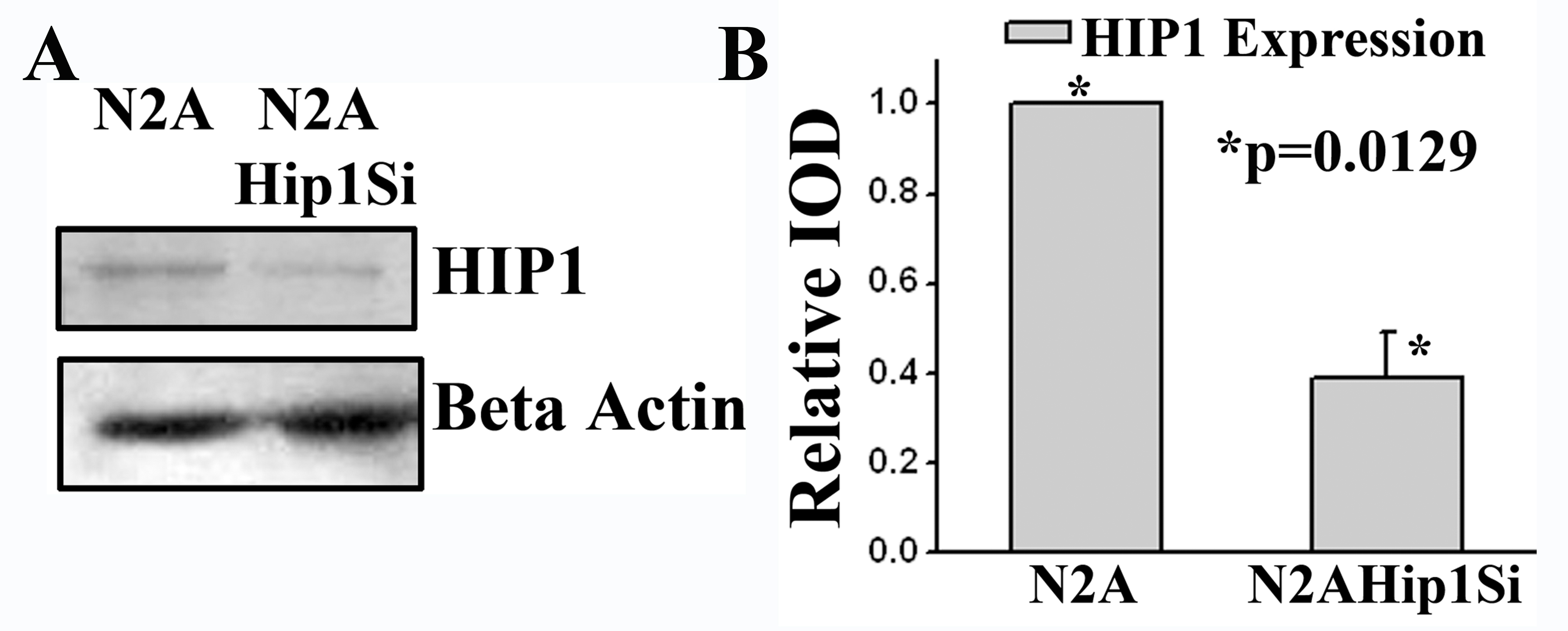

Supplement: Additional file 8 — Knock down of HIP1 in Neuro2A cells. A. Western blot analysis for the detection of HIP1 (upper panel) in Neuro2A cells (designated as N2A) and N2A cells stably transfected with Hip1Si construct to knock down HIP1 (designated as N2AHip1Si). In the lower panel, the 42 KDa band represents beta actin as loading control. B: The bar diagram represents mean integrated optical densities of bands corresponding to HIP1 and normalized with that of the loading control beta actin. Level of significance (p values) is shown in the figure. [file 1471-2164-12-463-S8.JPEG]
